# Supplementary material for: An integrated in vivo/in vitro framework to enhance cell-free biosynthesis with metabolically rewired yeast extracts
Source: Nat Commun. 2021 Aug 26;12:5139. doi: 10.1038/s41467-021-25233-y (PMC8390474; doi:10.1038/s41467-021-25233-y)
Supplement: Supplementary file 1 — Supplementary Information [file 41467_2021_25233_MOESM1_ESM.docx]

**Supplementary Information For**

**An integrated in vivo/in vitro framework to enhance cell-free biosynthesis with metabolically rewired yeast extracts**

Blake J. Rasor^a,b,c,^, Xiunan Yi^d^, Hunter Brown^a,b,c,^, Hal S. Alper^d,e,^*, and Michael C. Jewett^a,b,c,f,g,^*

1. Department of Chemical and Biological Engineering, Northwestern University, Evanston, IL 60208, USA
2. Chemistry of Life Processes Institute, Northwestern University, Evanston, IL 60208, USA
3. Center for Synthetic Biology, Northwestern University, Evanston, IL 60208, USA
4. Institute for Cellular and Molecular Biology, The University of Texas at Austin, Austin, TX, 78712, USA
5. McKetta Department of Chemical Engineering, The University of Texas at Austin, Austin, TX, 78712, USA
6. Robert H. Lurie Comprehensive Cancer Center, Northwestern University, Chicago, IL 60611, USA
7. Simpson Querrey Institute, Northwestern University, Chicago, IL 60611, USA

* Co-corresponding authors

**Correspondence**:

Hal Alper, [halper@che.utexas.edu](mailto:halper@che.utexas.edu)

Michael Jewett, [m-jewett@northwestern.edu](mailto:m-jewett@northwestern.edu)

**
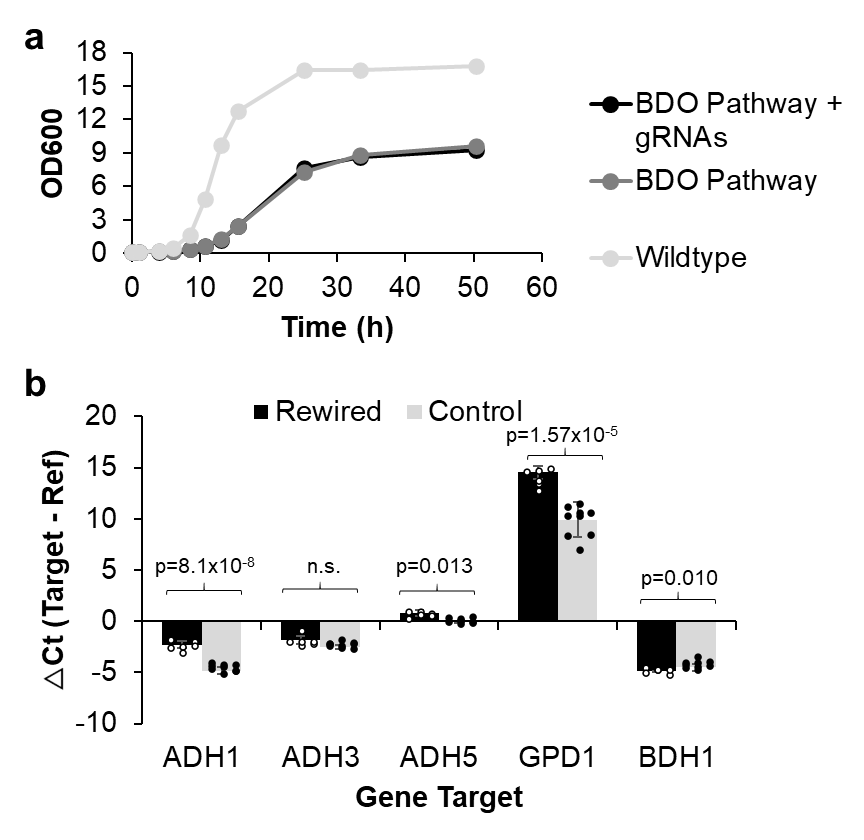
**

**Supp. Fig. 1.** Comparison of growth rates and gene expression. **a** Metabolic rewiring does not alter growth of BDO strain in 1 L cultures (n=1). **b** qPCR results for genes targeted by CRISPR effectors with 3 technical replicates from n=2-3 biological replicates. Higher ΔCt value indicates lower expression, and p-values were determined by a two-tailed Student’s t-test.


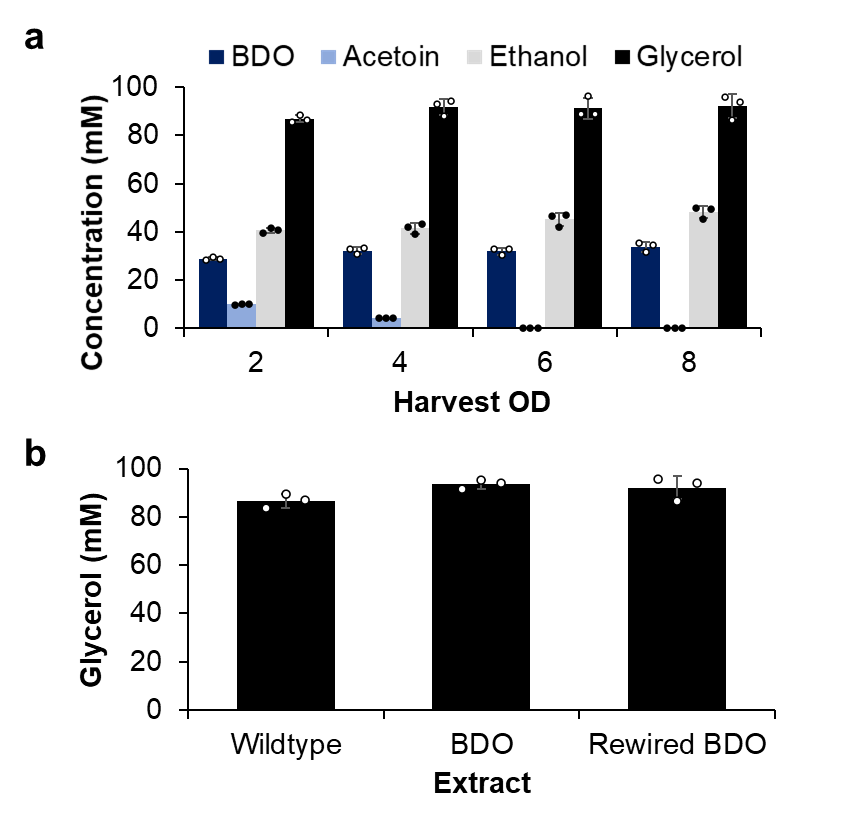


**Supp. Fig. 2.** Comparison of harvest OD and glycerol production. **a** Cell extracts from different phases of growth result in similar metabolite profiles. **b** Extracts from wildtype and engineered strains produce comparable glycerol concentrations. Data represent mean ± standard deviation of n=3 technical replicates.


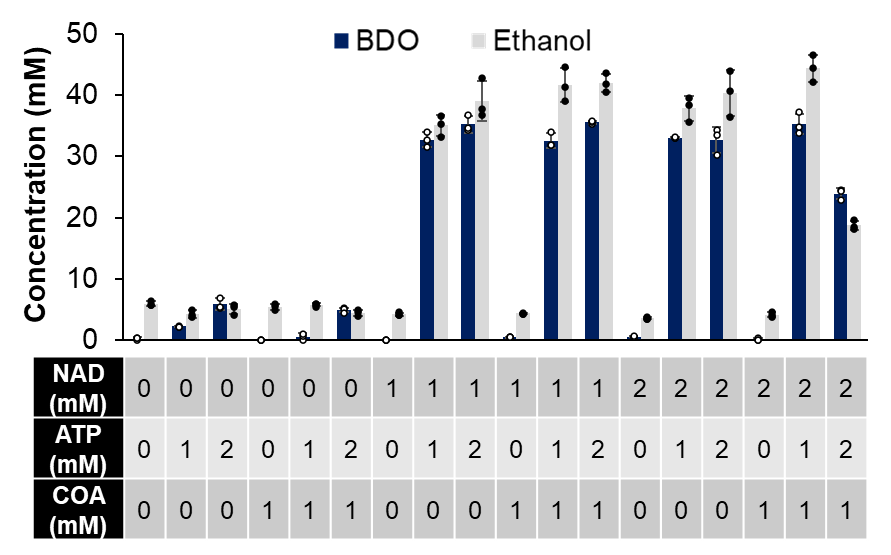


**Supp. Fig. 3.** In vivo BDO production. Yeast strains grown in 25 mL of YSC media within 250 mL shake flasks over 48-54 h produce BDO slowly as carbon flux is diverted to biomass with metabolically rewired strains consistently producing higher BDO titers. The rate of BDO production per mM glucose consumed is lower compared to in vitro results for similar stages of growth, although the culture conditions result in lower cell density at saturation. Data represent mean ± standard deviation of n=3 technical replicates.


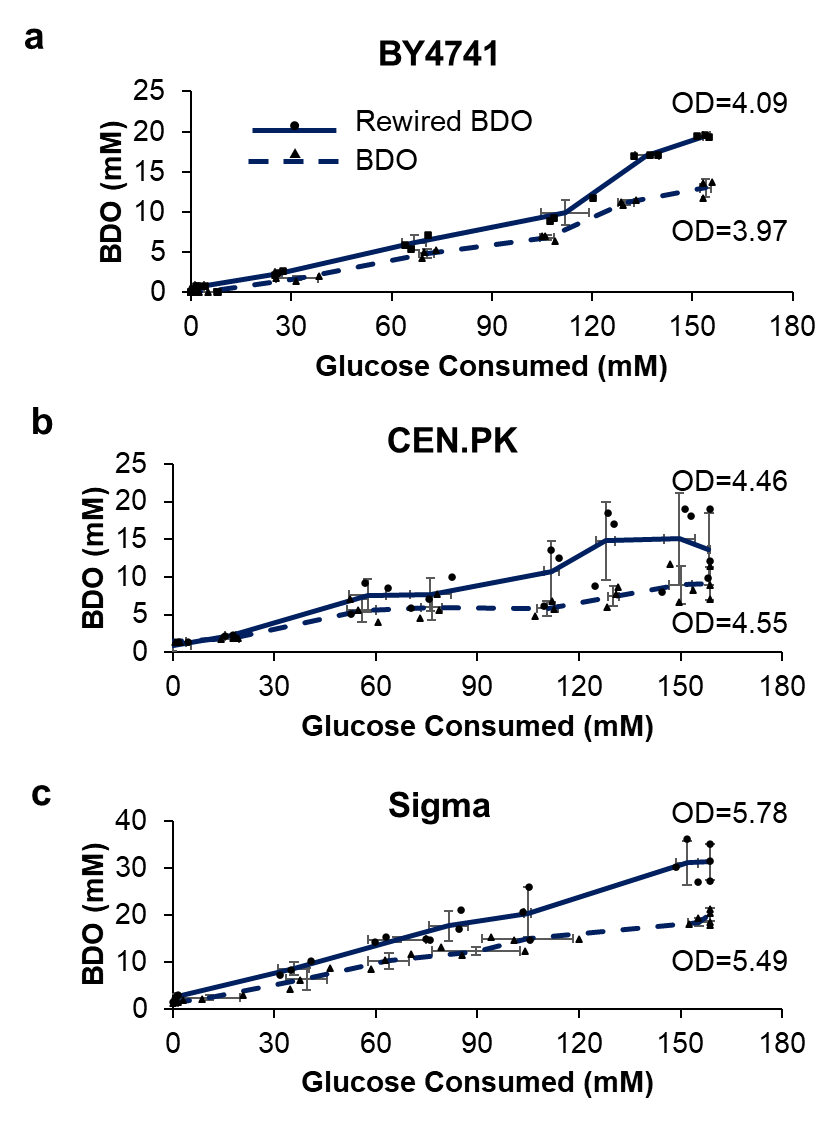


**Supp. Fig. 4.** In vivo BDO production. Yeast strains grown in 250 ml flasks over 48-54 h produce BDO slowly as carbon flux is diverted to biomass with metabolically rewired strains consistently producing higher BDO titers. The rate of BDO production per mM glucose consumed is lower compared to in vitro results for similar stages of growth, although the culture conditions result in lower cell density at saturation. Data represent mean ± standard deviation of n=3 biological replicates.


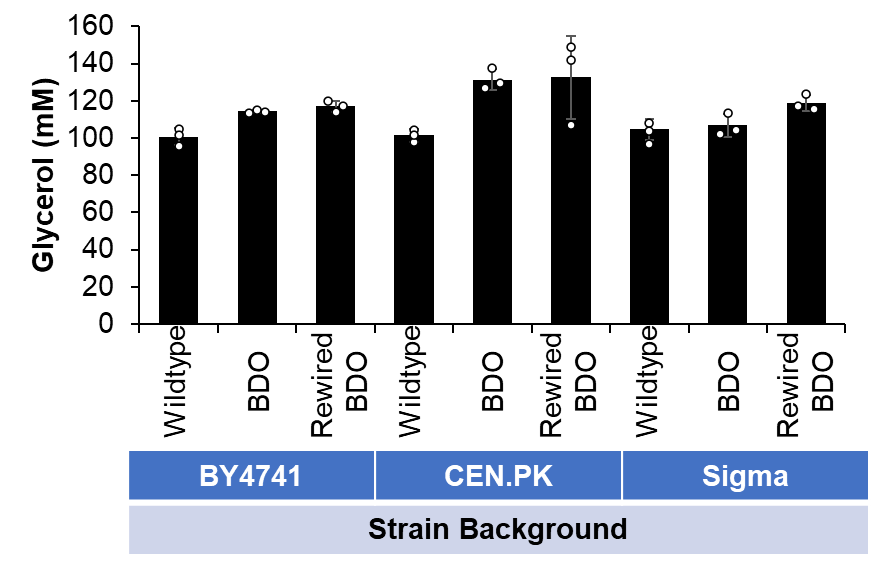


**Supp. Fig. 5.** Glycerol titers from BDO strain panel. Glycerol measurements are more consistent across different strains, making ethanol a more informative byproduct for comparison. Data represent mean ± standard deviation of n=3 technical replicates.


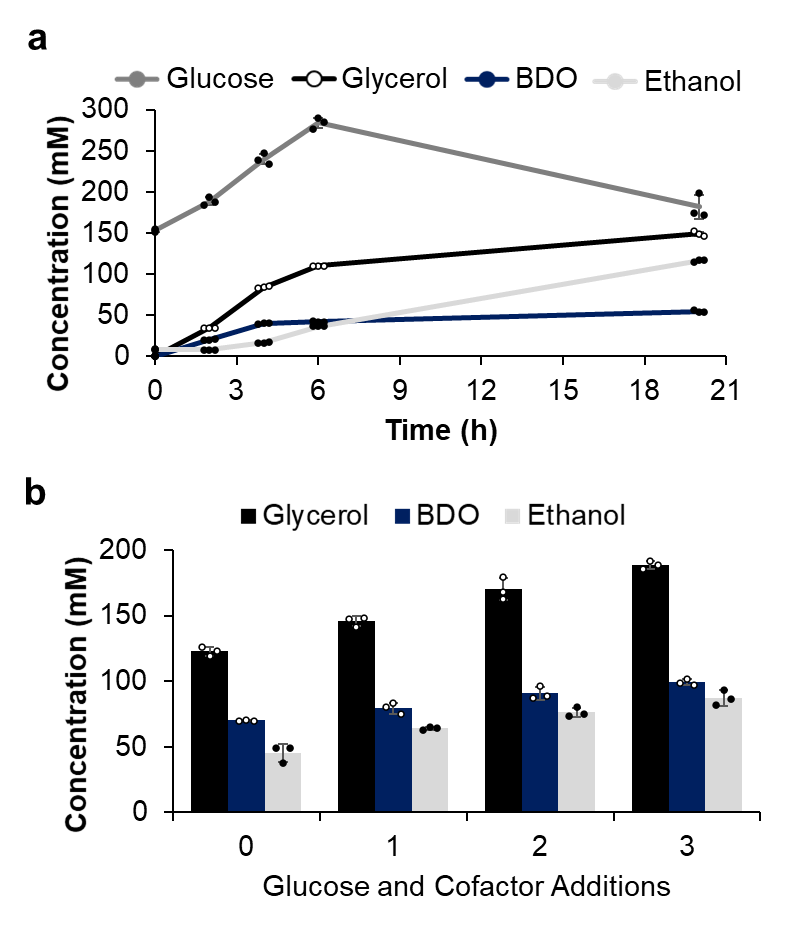


**Supp. Fig. 6.** Fed batch reaction optimization. **a** Feeding glucose alone at 2 h intervals results in decreased BDO titers due to reduced glucose consumption. **b** Supplementing 1 mM NAD and 2 mM ATP along with glucose additions increases BDO titer at 20 h as glucose is completely consumed. Data represent mean ± standard deviation of n=3 technical replicates.


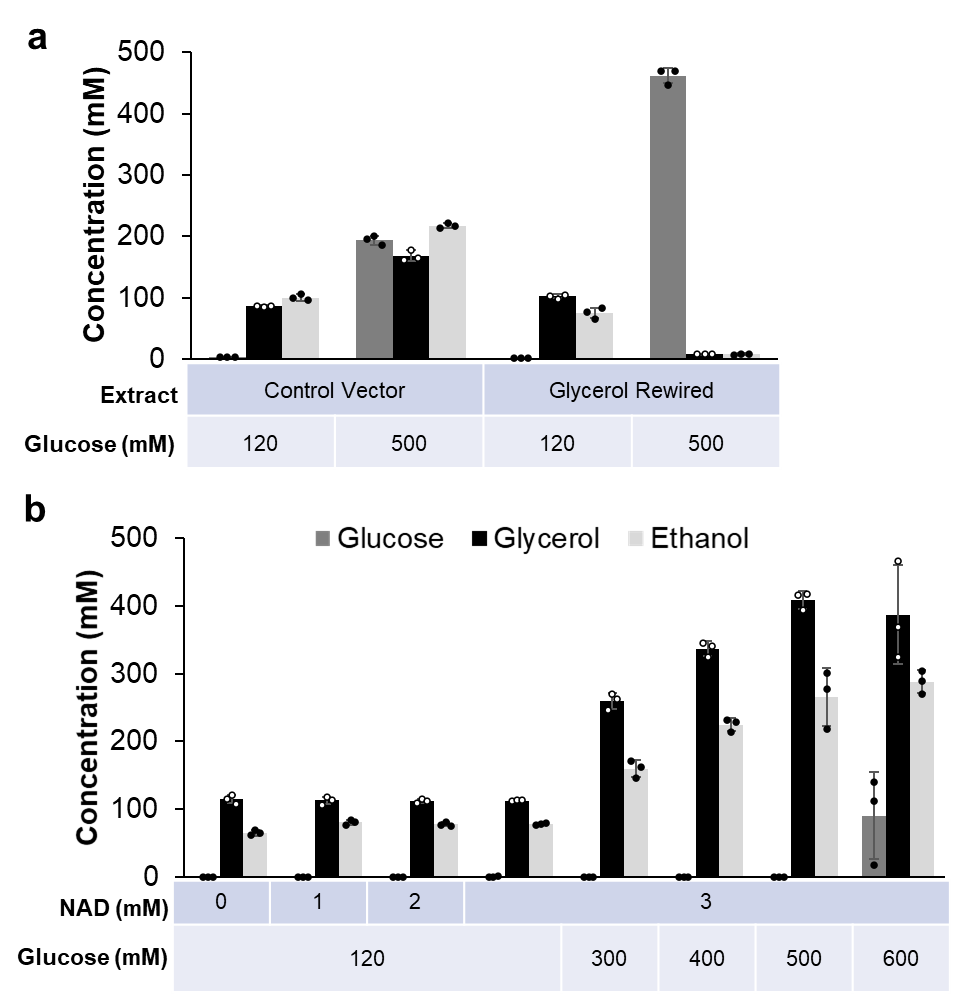


**Supp. Fig. 7.** Optimization of glycerol biosynthesis. **a** Metabolic rewiring via CRISPR effectors increased glycerol production ~20%, these extracts could not consume high concentrations of glucose. Extracts from strains modified by plasmid overexpression were utilized for further glycerol biosynthesis reactions. **b** Reactions were tuned to produce over 80% of the theoretical yield of glycerol by simultaneously increasing concentrations of glucose and NAD. Data represent mean ± standard deviation of n=3 technical replicates.


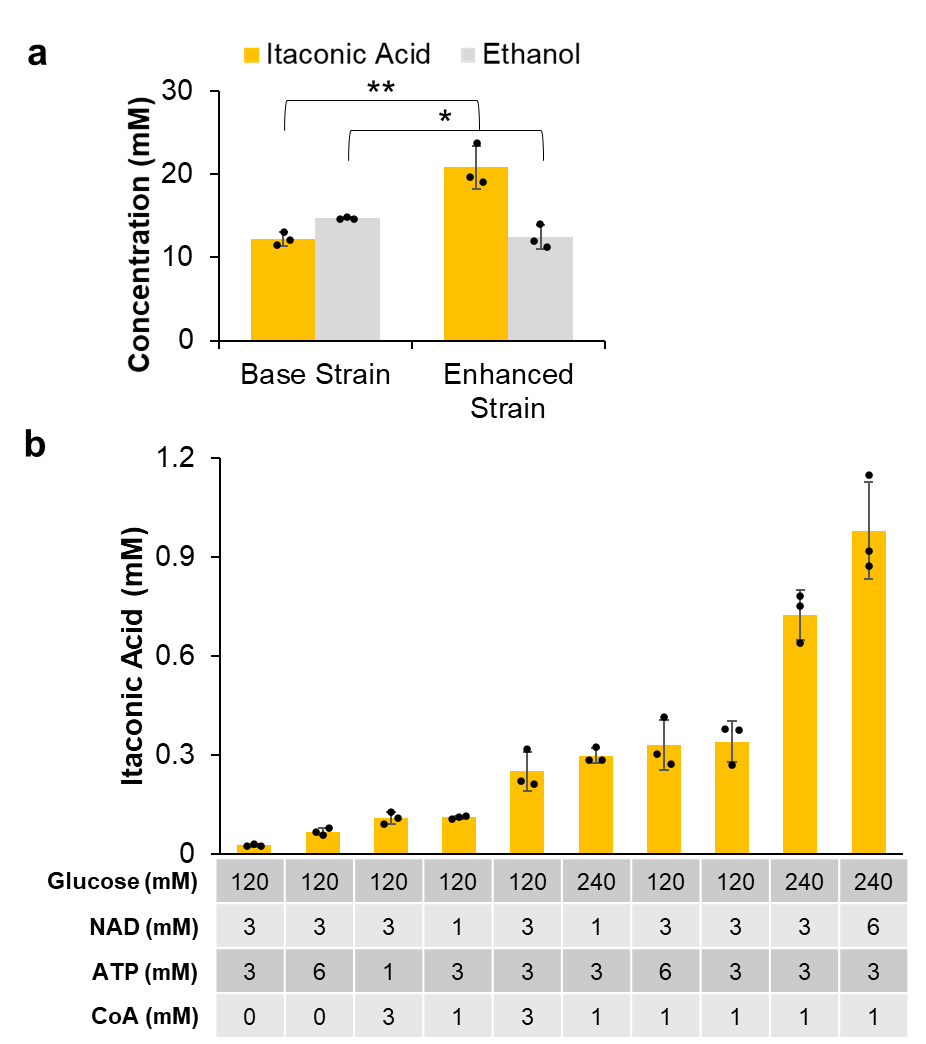


**Supp. Fig. 8.** Optimization of itaconic acid biosynthesis. **a** Reactions with 25 mM citrate as the substrate instead of glucose highlight the impact of strain engineering for this pathway and suggest that the low titers produced from glucose result from limited carbon flux into the TCA cycle. Extract from the enhanced strain produces more itaconic acid and less ethanol than extract from the base strain (**p = 0.0052 and *p = 0.054 as determined by a two-tailed Student’s t-test). **b** Cofactor optimization indicates NAD is key to increasing itaconic acid titers in cell-free reactions. Data represent mean ± standard deviation of n=3 technical replicates.

**Supp. Table 1. Yeast strains used in this study.** Pertinent information for each strain processed into cell extract, including the genetic background and plasmids encoding heterologous pathways and/or CRISPR-dCas9 effectors. “CV” denotes a control vector without sgRNAs to target dCas9 to genes for regulation.

| **Strain** | **Background** | **Plasmid(s)** | **Medium** | **Source** |
| --- | --- | --- | --- | --- |
| **Rewired BDO** | BY4741 | p416-(pFBA1-NoxE-IDP1t)-(pTPI1-AlsD-SPG5t)-(pPGK1-AlsS-PRM9t)  p415-(pTDH3-dCas9VPR-ADH1t)-sgRNA5 | CSM-Leu-Ura | This study |
| **Rewired BDO** | CEN.PK2-a |  |  | (Deaner et al., 2018) |
| **Rewired BDO** | Sigma 10560-4A |  |  |  |
| **BDO** | BY4741 | p416-(pFBA1-NoxE-IDP1t)-(pTPI1-AlsD-SPG5t)-(pPGK1-AlsS-PRM9t  p415-(pTDH3-dCas9VPR-ADH1t)-CV | CSM-Leu-Ura | This study |
| **BDO** | CEN.PK2-a |  |  | (Deaner et al., 2018) |
| **BDO** | Sigma 10560-4A |  |  |  |
| **Wildtype** | BY4741 | None | CSM | EUROSCARF |
| **Wildtype** | CEN.PK2-a |  |  |  |
| **Wildtype** | Sigma 10560-4A |  |  | Gerald Fink |
| **Itaconic acid base strain** | BY4741 his3::pGPD-CAD1-CYC1t-HIS3 | None | CSM-H | This study |
| **Itaconic acid enhanced strain** | BY4741 ΔADE3 ΔBNA2 | p416-Enhanced-pGPD-CAD1-CYC1t | CSM-U | (Blazeck *et al.*, 2014) |
| **Glycerol base strain** | BY4741 trp1::pGPD-dhaB-PRM9t-pTEF-yqhD-SPG5t | p415-pTEF-IDP1,  p414-pTDH3-GPDmcs2-PRM9t | CSM-WUL | (Deaner and Alper, 2017) |
| **Glycerol enhanced strain** |  | p415-pTEF-GPP1-IDP1t,  p414-pTDH3-GPDmcs2-GPD1-PRM9t |  |  |
| **BY4741 glycerol CRISPR control** | trp1::pGPD-dhaB-PRM9t-pTEF-yqhD-SPG5t | p415-THD3-dCas9VPR | CSM-UL |  |
| **BY4741 glycerol CRISPR rewired** |  | p415-THD3-dCas9VPR-SNR52-sgPD9-sgPD13 |  |  |

**Supp. Table 2. Cell extracts used in this study.** Strains in Supp. Table 1 were harvest at the listed cell density and processed into cell extracts as described in methods. The protein content for each extract was determined via Bradford assay (n=3 replicates for each of 3 dilution factors) to normalize cell-free reactions by protein concentration.

| **Designation** | **Background** | **Harvest OD_600_** | **Protein Content (mg/ml)** |
| --- | --- | --- | --- |
| **Rewired BDO** | BY4741 | 2.0 | 40.61 ± 7.31 |
| **Rewired BDO** | BY4741 | 4.04 | 42.24 ± 8.14 |
| **Rewired BDO** | BY4741 | 5.92 | 46.45 ± 8.55 |
| **Rewired BDO** | BY4741 | 7.90 | 36.83 ± 7.31 |
| **Rewired BDO** | CEN.PK2-a | 8.23 | 43.18 ± 4.70 |
| **Rewired BDO** | Sigma 10560-4A | 8.01 | 27.74 ± 3.48 |
| **BDO** | BY4741 | 7.90 | 33.79 ± 12.22 |
| **BDO** | CEN.PK2-a | 7.97 | 32.98 ± 3.83 |
| **BDO** | Sigma 10560-4A | 8.45 | 27.22 ± 3.16 |
| **Wildtype** | BY4741 | 8.0 | 40.08 ± 7.45 |
| **Wildtype** | CEN.PK2-a | 12.0 | 30.05 ± 10.10 |
| **Wildtype** | Sigma 10560-4A | 9.0 | 30.72 ± 4.31 |
| **Base itaconic acid** | BY4741 + his3::pGPD-CAD1-CYC1t-HIS3 | 8.61 | 16.77 ± 1.88 |
| **Enhanced itaconic acid** | BY4741 ΔADE3 ΔBNA2 | 5.40 | 19.16 ± 1.58 |
| **Base glycerol** | BY4741 trp1::pGPD-dhaB-PRM9t-pTEF-yqhD-SPG5t | 7.90 | 28.02 ± 10.16 |
| **Enhanced glycerol** |  | 7.03 | 17.27 ± 1.70 |
| **Glycerol CRISPR control** | BY4741 trp1::pGPD-dhaB-PRM9t-pTEF-yqhD-SPG5t | 5.67 | 30.79 ± 4.44 |
| **Glycerol CRISPR rewired** |  | 7.32 | 28.96 ± 3.57 |

**Supplementary Table 3. Biomass and metabolite concentrations in cultures or cell-free reactions over time.** Values show mean ± standard deviations of three (n = 3) independent experiments (biological replicates in vivo and technical replicates in vitro). Cell-free reactions contained 6 mg extract protein per mL, which corresponds to ~216 mg cell biomass per ml for this extract (1 g biomass yields ~1 mL of cell extract, and the rewired BDO Sigma extract contained 27.74 ± 3.48 mg protein / ml). Bolded rows represent the time at which significant product formation ceases in each system.

| **System** | **Time point (h)** | **Cell biomass (mg/ml)** | **BDO (mM)** |
| --- | --- | --- | --- |
| In vivo | 0 | 0.0407 ± 0 | 0 ± 0 |
| In vivo | 24 | 5.45 ± 0.22 | 10.37 ± 0.71 |
| **In vivo** | **48** | **9.42 ± 0.44** | **24.79 ± 1.28** |
| In vivo | 72 | 9.82 ± 0.44 | 24.95 ± 0.45 |
| Cell-free | 0 | 216 ± 27.57 | 0.57 ± 0.07 |
| **Cell-free** | **6** | **216 ± 27.57** | **62.43 ± 1.24** |
| Cell-free | 20 | 216 ± 27.57 | 70.46 ± 3.12 |

**Supplementary Table 4. Volumetric productivities normalized to cellular biomass.** Values show mean ± propagated error from three independent experiments. Bolded rows represent the time at which significant product formation ceases in each system.

| **System** | **Time point (h)** | **mM BDO / h** | **mmol BDO / h / g cell biomass** |
| --- | --- | --- | --- |
| In vivo | 24 | 0.432 ± 0 | 0.079 ± 0.0063 |
| **In vivo** | **48** | **0.516 ± 0.015** | **0.055 ± 0.0038** |
| In vivo | 72 | 0.346 ± 0.018 | 0.035 ± 0.0017 |
| **Cell-free** | **6** | **10.41 ± 0.011** | **0.048 ± 0.0062** |
| Cell-free | 20 | 3.523 ± 0.16 | 0.017 ± 0.0022 |

**Supp. Table 5. Guide RNA sequences used in this study.** Guide RNAs were used to regulate the expression of target targets. For repression, the guide RNA targets the ORF of genes (+) at the non-template strand. For activation, the guide RNA targets the promoter region of genes (-) at the template strand.

| **Targets** | **Regulation** | **Location (NGG Relative to ATG)** | **Sequences** |
| --- | --- | --- | --- |
| For BDO Rewiring | | | |
| ADH1 | Repression | +75 | AATTCGTTGGCCTTTGGCTT |
| ADH3 | Repression | +73 | AGTCTTAGGGATTGCAGCTG |
| ADH5 | Repression | +84 | ATTTCGTTAGGCTTAGGTTC |
| GPD1 | Repression | +96 | CCAATCACAGTAACCTTGAA |
| BDH1 | Activation | -207 | CCTATTCTTTCCTCCTTACG |
| For Glycerol Rewiring | | | |
| GPD1 | Activation | -178 | AACCTAATTCGCACGTAGAC |
| GPP1 | Activation | -427 | ATTGCAGGATTCTCATTGTC |

**Supp. Table 6. Accessory sequences for sgRNA constructs.** To rewire the cells for improved BDO production, a sgRNA cassette with tRNA-sgRNA bricks was used. The sequences of tRNAs and the whole cassette is as below. The expression is under the control of TEF1 promoter.

| **Cassettes** | **Sequences** |
| --- | --- |
| RNA Scaffold | GTTTTAGAGCTAGAAATAGCAAGTTAAAATAAGGCTAGTCCGTTATCAACTTGAAAAAGTGGCACCGAGTCGGTGCTTTT |
| tRNA (tTCT) | GCTCGCGTGGCGTAATGGCAACGCGTCTGACTTCTAATCAGAAGAtTATGGGTTCGACCCCCATCGTGAGTG |
| tRNA (tCTT) | GCCTTGTTGGCGCAATCGGTAGCGCGTATGACTCTTAATCATAAGGtTAGGGGTTCGAGCCCCCTACAGGGCT |
| tRNA (tGTT) | GACTCCATGGCCAAGTTGGTtAAGGCGTGCGACTGTTAATCGCAAGAtCGTGAGTTCAACCCTCACTGGGGTCG |
| tRNA (tGTC) | TCCGTGATAGTTTAATGGTcAGAATGGGCGCTTGTCGCGTGCCAGaTCGGGGTTCAATTCCCCGTCGCGGAG |
| tRNA (tGCC) | GCGCAAGTGGTTTAGTGGTAAAATCCAACGTTGCCATCGTTGGGCCCCCGGTTCGATTCCGGGCTTGCGCA |
| tRNA (tTTC) | TCCGATATAGTGTAACGGCtATCACATCACGCTTTCACCGTGGAGaCCGGGGTTCGACTCCCCGTATCGGAG |
| Whole sgRNA Cassette for BDO Rewiring | ACTAGTAGTAGCTCGCGTGGCGTAATGGCAACGCGTCTGACTTCTAATCAGAAGAtTATGGGTTCGACCCCCATCGTGAGTGCCTATTCTTTCCTCCTTACGGTTTTAGAGCTAGAAATAGCAAGTTAAAATAAGGCTAGTCCGTTATCAACTTGAAAAAGTGGCACCGAGTCGGTGCTTTTGCTAGTAATAGCCTTGTTGGCGCAATCGGTAGCGCGTATGACTCTTAATCATAAGGtTAGGGGTTCGAGCCCCCTACAGGGCTCCAATCACAGTAACCTTGAAGTTTTAGAGCTAGAAATAGCAAGTTAAAATAAGGCTAGTCCGTTATCAACTTGAAAAAGTGGCACCGAGTCGGTGCTTTTGCTAGTAAGTGACTCCATGGCCAAGTTGGTtAAGGCGTGCGACTGTTAATCGCAAGAtCGTGAGTTCAACCCTCACTGGGGTCGATTTCGTTAGGCTTAGGTTCGTTTTAGAGCTAGAAATAGCAAGTTAAAATAAGGCTAGTCCGTTATCAACTTGAAAAAGTGGCACCGAGTCGGTGCTTTTGCTAGTTTCTTCCGTGATAGTTTAATGGTcAGAATGGGCGCTTGTCGCGTGCCAGaTCGGGGTTCAATTCCCCGTCGCGGAGAGTCTTAGGGATTGCAGCTGGTTTTAGAGCTAGAAATAGCAAGTTAAAATAAGGCTAGTCCGTTATCAACTTGAAAAAGTGGCACCGAGTCGGTGCTTTTGCTAGTATAAGCGCAAGTGGTTTAGTGGTAAAATCCAACGTTGCCATCGTTGGGCCCCCGGTTCGATTCCGGGCTTGCGCAAATTCGTTGGCCTTTGGCTTGTTTTAGAGCTAGAAATAGCAAGTTAAAATAAGGCTAGTCCGTTATCAACTTGAAAAAGTGGCACCGAGTCGGTGCTTTTGCTAGTATCATCCGATATAGTGTAACGGCtATCACATCACGCTTTCACCGTGGAGaCCGGGGTTCGACTCCCCGTATCGGAG |

**Supp. Table 7. Primers for qPCR used in this study.**

| **Target** | **Direction** | **Sequences** |
| --- | --- | --- |
| ADH1 | Forward | TATCTTCTACGAATCCCACGG |
|  | Reverse | CTTTGGCTTTGGAACTGGA |
| ADH3 | Forward | GCCATTACCTGTTAAACTACCA |
|  | Reverse | TTTGACAACTACACCAGCAC |
| ADH5 | Forward | CGTTAAGGGCTGGAAAGTC |
|  | Reverse | CATGCAAGTCCCATTCAACC |
| BDH1 | Forward | CTAATCACTGGTAAGCAAAGGA |
|  | Reverse | CCATCAACTCTTGGAATCCC |
| GPD1 | Forward | AAGTTCACGAATGGTTGGA |
|  | Reverse | ACGGCTTCAAATAATGGGA |
| ALG9 (Reference) | Forward | GCTCCTATAGCCGTCTACGAGC |
|  | Reverse | CTGGCAGCAGGAAAGAACTTGG |
