# Supplementary material for: An integrated in vivo/in vitro framework to enhance cell-free biosynthesis with metabolically rewired yeast extracts
Source: Nat Commun. 2021 Aug 26;12:5139. doi: 10.1038/s41467-021-25233-y (PMC8390474; doi:10.1038/s41467-021-25233-y)
Supplement: Supplementary file 2 — Reporting Summary [file 41467_2021_25233_MOESM2_ESM.pdf]

nature research

Corresponding author(s): Hal Alper and Michael Jewett

Last updated by author(s): 7-27-2021

Reporting Summary

Nature Research wishes to improve the reproducibility of the work that we publish. This form provides structure for consistency and transparency in reporting. For further information on Nature Research policies, see our Editorial Policies and the Editorial Policy Checklist.

Statistics

For all statistical analyses, confirm that the following items are present in the figure legend, table legend, main text, or Methods section.

n/a	Confirmed	
☐	☒	The exact sample size (n) for each experimental group/condition, given as a discrete number and unit of measurement
☐	☒	A statement on whether measurements were taken from distinct samples or whether the same sample was measured repeatedly
☐	☒	The statistical test(s) used AND whether they are one- or two-sided<br>*Only common tests should be described solely by name; describe more complex techniques in the Methods section.*
☐	☒	A description of all covariates tested
☒	☐	A description of any assumptions or corrections, such as tests of normality and adjustment for multiple comparisons
☐	☒	A full description of the statistical parameters including central tendency (e.g. means) or other basic estimates (e.g. regression coefficient) AND variation (e.g. standard deviation) or associated estimates of uncertainty (e.g. confidence intervals)
☐	☒	For null hypothesis testing, the test statistic (e.g. F, t, r) with confidence intervals, effect sizes, degrees of freedom and P value noted<br>*Give P values as exact values whenever suitable.*
☒	☐	For Bayesian analysis, information on the choice of priors and Markov chain Monte Carlo settings
☒	☐	For hierarchical and complex designs, identification of the appropriate level for tests and full reporting of outcomes
☒	☐	Estimates of effect sizes (e.g. Cohen's d, Pearson's r), indicating how they were calculated

Our web collection on statistics for biologists contains articles on many of the points above.

Software and code

Policy information about availability of computer code

Data collection	All data were collected using instruments identified in the methods and associated, commercially available software. No in-house algorithms or softwares were used to collect the data. Commercial software used includes: Agilent ChemStation (revision B.04.03[16]) for HPLC data, BioTek Gen5 (version 2.09) for Bradford assays, ThermoFisher Scientific NanoDrop 2000c (version 1.6.198) for optical density measurements, and PrimerBLAST (https://www.ncbi.nlm.nih.gov/tools/primer-blast/) for primer sequence generation.
Data analysis	All data were analyzed using commercially-available software. No in-house algorithms or software were used to analyze and display the data. Software for data analysis includes: Excel (Microsoft Office 16, version 2106 build 14131.20332) and Adobe Illustrator 2021 (version 25.0.1)

For manuscripts utilizing custom algorithms or software that are central to the research but not yet described in published literature, software must be made available to editors and reviewers. We strongly encourage code deposition in a community repository (e.g. GitHub). See the Nature Research guidelines for submitting code & software for further information.

Data

Policy information about availability of data

All manuscripts must include a data availability statement. This statement should provide the following information, where applicable:

- Accession codes, unique identifiers, or web links for publicly available datasets
- A list of figures that have associated raw data
- A description of any restrictions on data availability

All data generated and analyzed for this study are presented in the manuscript or supplementary information or are available upon request from the corresponding author. There are no restrictions on data availability, and the source data are provided with this publication.

Field-specific reporting

Please select the one below that is the best fit for your research. If you are not sure, read the appropriate sections before making your selection.

[x] Life sciences [ ] Behavioural & social sciences [ ] Ecological, evolutionary & environmental sciences

For a reference copy of the document with all sections, see nature.com/documents/nr-reporting-summary-flat.pdf

Life sciences study design

All studies must disclose on these points even when the disclosure is negative.

Sample size	All in vitro experiments used n=3 replicates of unique cell-free reactions, as is regular practice in cell-free literature. Time course data for cell-free reactions used individual samples for each time point, as the analytical methods utilized could not be performed continuously. In vivo measurements incorporated n=3 biological replicates. Sample sizes were determined based on precedent in previous literature (not by calculations) to enable statistical analyses such as standard deviation and t-tests.
Data exclusions	None to report.
Replication	All attempts at replication were successful and technical triplicates of each sample were used to verify reproducibility. Biological replicates of cell extracts had reproducible responses to methodological variables optimized in the manuscript in at least 2 independent experiments.
Randomization	Samples were analyzed equally, then characterized fully and reported in completion. There was no requirement for randomization due to the controlled chemical nature of cell-free systems.
Blinding	Samples were designated with letters prior to HPLC analysis to ensure unbiased evaluation of metabolite concentrations. No other blinding was necessary.

Reporting for specific materials, systems and methods

We require information from authors about some types of materials, experimental systems and methods used in many studies. Here, indicate whether each material, system or method listed is relevant to your study. If you are not sure if a list item applies to your research, read the appropriate section before selecting a response.

Materials & experimental systems

n/a	Involved in the study	
[x]	[ ]	Antibodies
[x]	[ ]	Eukaryotic cell lines
[x]	[ ]	Palaeontology and archaeology
[x]	[ ]	Animals and other organisms
[x]	[ ]	Human research participants
[x]	[ ]	Clinical data
[x]	[ ]	Dual use research of concern

Methods

n/a	Involved in the study	
[x]	[ ]	ChIP-seq
[x]	[ ]	Flow cytometry
[x]	[ ]	MRI-based neuroimaging
